# Danish Medical Statistics

**Published:** 1841-01

**Authors:** 


*Danish Medical Statistics.*—In the Danish Medical Library for January, February and March, of the present year, there are copious extracts from the Transactions of the Royal Society of Health for 1839. The first is an account of the prevailing diseases in Denmark, with the exception of Laaland and Falster, during the year 1838. These are smallpox, scarlet fever, measles, catarrhal complaints, typhus, which has been very prevalent, rheumatic fever, puerperal fever, psora, syphilis, croup and hooping cough, which last, after an absence of six years, has re-appeared among persons of all ages in the Faroe Isles. We are also informed that in the same year (1838) 18,813 persons were vaccinated in the whole kingdom for the first time, and 1052 were re-vaccinated. That in Iceland 481 were vaccinated for the first time, and 647 were re-vaccinated.

The tables of births and deaths are not very precise. The number of accidental deaths and suicides, strangely enough united together, was 514.—*Bibliothek fur Læger. Boston Med. and Sur. Jour. for Dec.* 1840.

